# Comprehensive characterization of putative genetic influences on plasma metabolome in a pediatric cohort

**DOI:** 10.1186/s40246-022-00440-w

**Published:** 2022-12-08

**Authors:** In-Hee Lee, Matthew Ryan Smith, Azam Yazdani, Sumiti Sandhu, Douglas I. Walker, Kenneth D. Mandl, Dean P. Jones, Sek Won Kong

**Affiliations:** 1 Computational Health Informatics Program, Boston Children’s Hospital, 401 Park Drive, Boston, MA 02215 USA; 2 Division of Pulmonary, Allergy, and Critical Care Medicine, Department of Medicine, Emory University, Atlanta, GA 30602 USA; 3 Atlanta Department of Veterans Affairs Medical Center, Decatur, GA 30033 USA; 4 Center of Perioperative Genetics and Genomics, Department of Anesthesiology, Perioperative and Pain Medicine, Brigham and Women’s Hospital, Harvard Medical School, Boston, MA 02115 USA; 5 Department of Environmental Medicine and Public Health, Icahn School of Medicine at Mount Sinai, New York, NY 10029 USA; 6 Department of Biomedical Informatics, Harvard Medical School, Boston, MA 02115 USA; 7 Department of Pediatrics, Harvard Medical School, Boston, MA 02115 USA

## Abstract

**Background:**

The human exposome is composed of diverse metabolites and small chemical compounds originated from endogenous and exogenous sources, respectively. Genetic and environmental factors influence metabolite levels, while the extent of genetic contributions across metabolic pathways is not yet known. Untargeted profiling of human metabolome using high-resolution mass spectrometry (HRMS) combined with genome-wide genotyping allows comprehensive identification of genetically influenced metabolites. As such previous studies of adults discovered and replicated genotype–metabotype associations. However, these associations have not been characterized in children.

**Results:**

We conducted the largest genome by metabolome-wide association study to date of children (*N* = 441) using 619,688 common genetic variants and 14,342 features measured by HRMS. Narrow-sense heritability (*h*^2^) estimates of plasma metabolite concentrations using genomic relatedness matrix restricted maximum likelihood (GREML) method showed a bimodal distribution with high *h*^2^ (> 0.8) for 15.9% of features and low *h*^2^ (< 0.2) for most of features (62.0%). The features with high *h*^2^ were enriched for amino acid and nucleic acid metabolism, while carbohydrate and lipid concentrations showed low *h*^2^. For each feature, a metabolite quantitative trait loci (mQTL) analysis was performed to identify genetic variants that were potentially associated with plasma levels. Fifty-four associations among 29 features and 43 genetic variants were identified at a genome-wide significance threshold *p* < 3.5 × 10^–12^ (= 5 × 10^–8^/14,342 features). Previously reported associations such as *UGT1A1* and bilirubin; *PYROXD2* and methyl lysine; and *ACADS* and butyrylcarnitine were successfully replicated in our pediatric cohort. We found potential candidates for novel associations including *CSMD1* and a monostearyl alcohol triglyceride (*m/z* 781.7483, retention time (RT) 89.3 s); *CALN1* and Tridecanol (*m/z* 283.2741, RT 27.6). A gene-level enrichment analysis using MAGMA revealed highly interconnected modules for dADP biosynthesis, sterol synthesis, and long-chain fatty acid transport in the gene-feature network.

**Conclusion:**

Comprehensive profiling of plasma metabolome across age groups combined with genome-wide genotyping revealed a wide range of genetic influence on diverse chemical species and metabolic pathways. The developmental trajectory of a biological system is shaped by gene–environment interaction especially in early life. Therefore, continuous efforts on generating metabolomics data in diverse human tissue types across age groups are required to understand gene–environment interaction toward healthy aging trajectories.

**Supplementary Information:**

The online version contains supplementary material available at 10.1186/s40246-022-00440-w.

## Background

Metabolites are indicators and effectors of biological processes that are controlled by genetic and environmental factors; thus, metabolite levels reflect homeostatic and pathological status [1]. Furthermore, metabolites are the regulators of epigenetic modification, gene expression, and protein activity [2]. Comprehensive profiling of metabolites in an individual—a metabotype—can provide a snapshot of the host genetic makeup and its interaction with diet and environmental exposures. Genetic variants affect metabolite levels by regulating gene expression and/or changing protein function in metabolic pathways. A single base change in DNA sequence can have a strong impact on metabolite concentrations in patients with an inborn error of metabolism (IEM); examining perturbed metabolic pathways helps elucidate the molecular pathophysiology of human diseases and therapeutic targets [3]. Furthermore, metabolite levels can be used as endophenotype that mediates genetic risks for common diseases [4] and predict inter-individual differences in drug response that are associated with pharmacogenetic variations [5].

Endogenous metabolite levels are highly heritable [6, 7]; genetically influenced metabotypes (GIMs) can be discerned with targeted or untargeted metabolomic profiling combined with genome-wide genotyping [8–11]. Using a genome-wide association study (GWAS) framework, a recent meta-analysis confirmed the reported associations between single nucleotide variants (SNVs) and metabotypes from independent cohort studies of adults [12]. The human metabolome is likely to contain hundreds of thousands of chemicals [13]; however, the breadth of chemical space coverage was limited up to few hundreds in previous studies. Therefore, the full extent of GIMs in the human metabolome is not yet known.

Untargeted high-resolution metabolomics (HRM) platforms enable quantitative measurements for tens of thousands of features with mass-to-charge ratios (*m/z*) with retention times (in seconds; RT) from endogenous and exogenous origins in biospecimens [3, 14]. A liquid chromatography high-resolution mass spectrometry (LC-HRMS) platform combined with genome-wide genotyping can provide a comprehensive snapshot of GIMs. With this platform, we previously evaluated the coverage of chemical space and constructed a global correlation map of the human plasma metabolome, measuring metabolites produced by the gut microbiome and xenobiotics and finding that many metabolites were associated with demographic characteristics [15].

In the current study, we deployed an untargeted LC-HRMS platform to analyze plasma samples collected from a pediatric cohort (*N* = 441) for which common genetic variants were characterized using genome-wide genotyping microarray. By interrogating 14,342 features and 619,688 common genetic variants, we first estimated narrow-sense heritability for all features. Further we performed a genome by metabolome-wide association study (GxMWAS) to replicate previously reported GIMs in our pediatric cohort and to discover novel GIM candidates after controlling for age, gender, and global genetic ancestry. Our results clearly revealed the extent of genetic contribution to metabotype across age groups for a wide range of chemical species.

## Results

### Chemical space coverage and the features associated with demographic factors

**Fig. 1:**
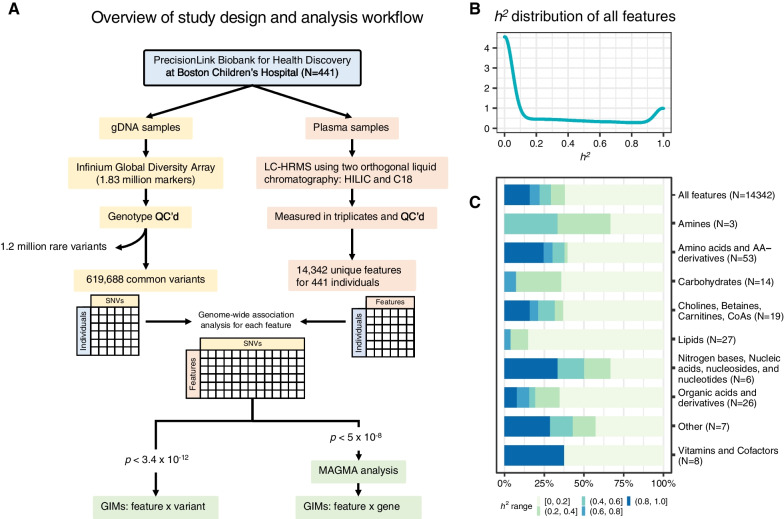
Overview of integrative analysis workflow and narrow-sense heritability estimates. **A** Data generation and analysis workflow. **B** Overall distribution of the narrow-sense heritability (*h*^2^) for all features. **C** Composition of features by their *h*^2^ values across chemical classes. The *h*^2^ values are binned into five groups by 0.2. All features’ group (top) includes all 14,342 features. Only the identified metabolites are grouped under each of the 9 categories

Samples, data generation, and analysis workflow are depicted in Fig. 1A. All LC-HRMS analysis was performed in triplicate using a dual column chromatography scheme that included hydrophilic interaction liquid chromatography (HILIC) and reversed phase liquid chromatography (RPLC; C18) columns to maximize the chemical space coverage. A total of 14,342 features were quantitatively measured with accurate *m/z* and RT for HILIC and C18 columns (*N* = 8739 and 5603, respectively). All features were subjected for metabolite annotation using xMSannotator [16], and high or medium confidence annotations at a 5-ppm mass tolerance window were used to reduce incorrect annotations (see Methods). A subset of features was experimentally identified using LC–MS/MS with authentic standards (*N* = 97 for HILIC and 69 for C18, Additional file 2: Table S1) [17]. The demographic characteristics of our cohort are summarized in Additional file 3: Table S2. To prioritize metabolites that were associated with demographic variables, we used a generalized linear model while controlling for batch effect and global genetic ancestry using the top ten eigenvectors—hereafter referred to as principal components (PCs) 1–10—from EIGENSTRAT analysis of 619,688 common variants (see Additional file 1: Fig. S1 for population stratification with the first two PCs).

A total of 338 features were associated with age and gender (false discovery rate (FDR) < 0.05, *N* = 120 and 218, respectively), of which 19 were among the identified metabolites using reference standards (*N* = 9 and 10, respectively; Additional file 4: Table S3). Among the age-correlated features, urate and creatinine are known to be correlated with age [18, 19] and we found novel associations including cystine, trimethyllysine, quinic acid, butyrobetaine, arachidonic acid, and retinol. Between females and males, amino acid derivatives (hydroxyproline, hydroxylysine, dimethylarginine), carnitine, cholines ((lysoPC(18:0) and lysoPC(18:1)), microbial product (valerobetaine), and a product of urea cycle (fumaric acid) were significantly different among the identified metabolites.

As of the impact of global genetic ancestry on individual’s metabotype, PC1 that distinguishes African descents from non-African descents was correlated with 141 features including 8 identified metabolites: amino acid derivatives (citrulline, indoleacetate, and kynurenine), carbohydrate (arabinose), vitamins (thiamine (B1), nicotinamide (B3)), and cholines (lysoPE(18:0), lysoPE(20:3)) (FDR < 0.05) (Additional file 4: Table S3). Except for PC4 and PC7, the other PCs were not correlated with any of the identified metabolites. PC4 was correlated with subgroups of European decent and PC7 distinguished two individuals of European descents. Nonetheless, the features that were significantly correlated with PCs may reflect combined effect of genetic and environmental factors, such as diet and lifestyle. We did not observe any bias in *m/z* and RT for the significant features associated with demographic variables—age, gender, and global genetic ancestry (Additional file 1: Figs. S2–S4).

Next, we performed pathway enrichment analysis using Mummichog [20] with statistical scores obtained from univariate analyses as described above. Pathways were selected for adjusted *p* value < 0.01 with ≥ 5 identified metabolites or uniquely annotated features (i.e., level 2 according to Schymanski et al. [21], see Methods) overlapping for each pathway (Additional file 5: Table S4). Glutamate metabolism pathway including five identified metabolites—glutamine, glutamate, carbamoyl phosphate, 2-oxoglutarate, and *N*-methylglycine—was significant for age (adjusted *p* value 0.00495). Tricarboxylic acid (TCA) cycle pathway was enriched with differentially detected metabolites between males and females. Ascorbate and aldarate metabolism and pentose phosphate pathways were significant for PC1 and PC4.

### Narrow-sense heritability estimation of feature levels

To capture the genetic influence on the variance of metabolite levels across individuals, we estimated a narrow-sense heritability (*h*^2^) of feature levels using genomic relatedness matrix (GRM) restricted maximum likelihood (GREML) method implemented in genome-wide complex trait analysis (GCTA) [22]. We found a wide range of *h*^2^ estimates with a bimodal distribution having 15.9% of features with high *h*^2^ (> 0.8) and 62.0% with low *h*^2^ (< 0.2) **(**Fig. 1B). Next, we checked *h*^2^ distributions in each chemical species with the identified metabolites. Carbohydrates and lipids had low *h*^2^ overall with 7.1% and 3.7% had *h*^2^ between 0.6–0.8, respectively. No feature was high *h*^2^ (> 0.8) for these chemical classes. In comparison, large proportions of amino acids and derivatives and nucleic acids had high *h*^2^ (> 0.8) (24.5% and 33.3%, respectively) (Fig. 1C).

### Genome by metabolome-wide association analysis

**Fig. 2:**
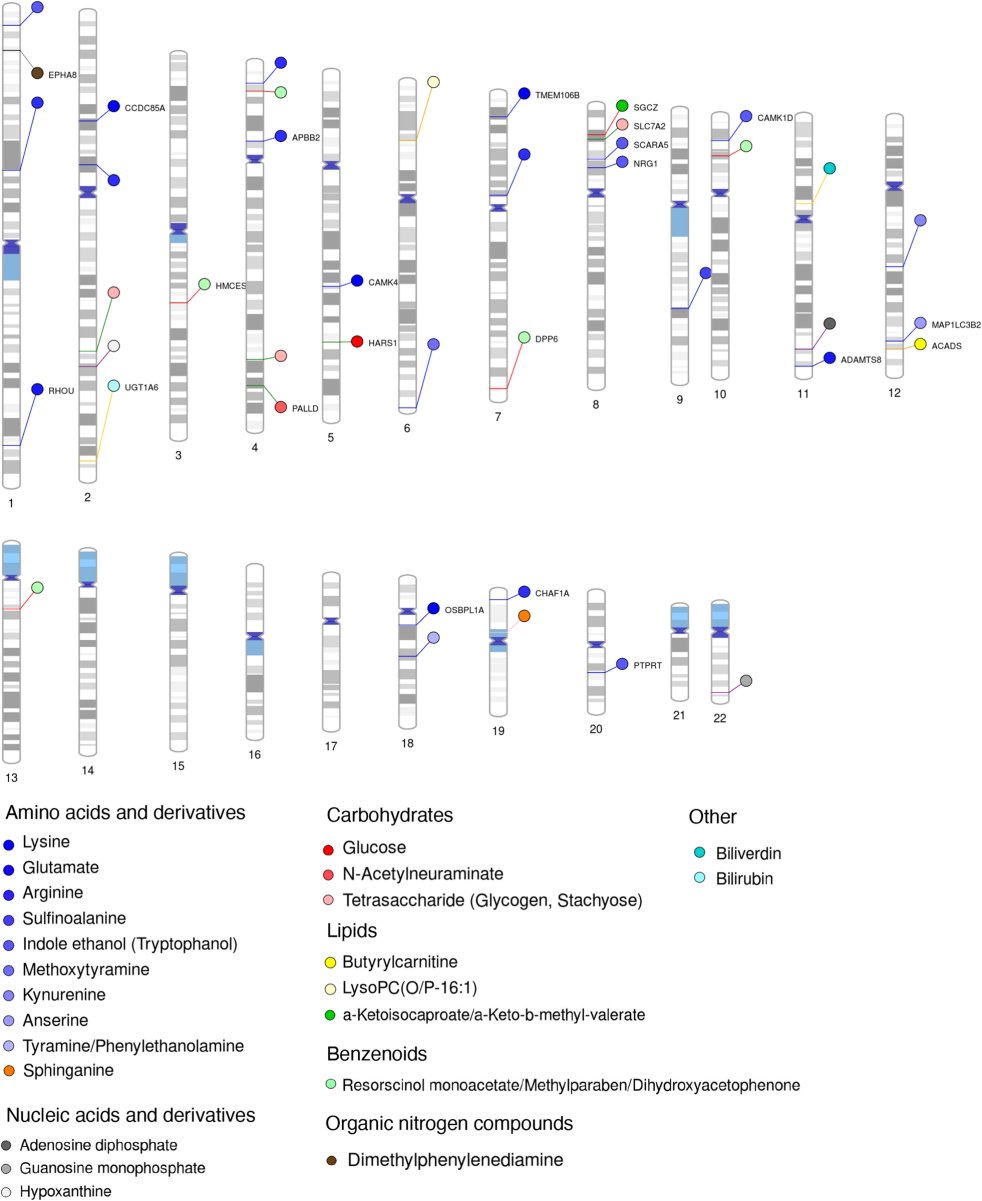
Genetically influenced metabotypes for identified metabolites. Each association is represented by identified metabolite (circles) pointing genetic variants along the genome (*p* < 5 × 10^–8^). Gene name is shown for significant loci next to the circle

We calculated age and gender corrected feature intensities and included the top 10 PCs as covariates to perform GxMWAS (see Methods) discovering 54 associations among 29 features and 43 common genetic variants at the threshold of *p* < 3.5 × 10^–12^ (= 5 × 10^–8^/14,342 features) (Table 1 for selected associations; Fig. 2 for associations involving identified metabolites with *p* < 5 × 10^–8^; Additional file 6: Table S5 for full list of associations at genome-wide significance level, *p* < 5 × 10^–8^; Additional file 7: Table S6 for full list of high and medium confidence annotations at 5-ppm mass tolerance). On average, a genetic variant was associated with 1.3 ± 0.79 features (range 1–4) and a feature was associated with the median of one variant (range 1–5). Most variants associated with feature levels were intronic (25 of 43, 58.1%) and three (7.0%) were in protein coding exons.

**Table 1:** Genetically influenced metabolites. Significant genetic variant–metabolite associations at a genome-wide significance of *p* < 3.5 × 10^–12^ (= 5 × 10^–8^/14,342 features) are shown.

Feature (*m/z*, RT)	Lead SNP position*	EA, OA	EAF	Beta	Standard error	*p* value	Candidate genes
*m/z* 394.8915, RT 68.1	chr9:72,902,395	G, A	0.14	− 0.49	0.063	4.94E−14	*ALDH1A1*
*m/z* 640.3195, RT 278.2	chr20:38,052,517	G, T	0.08	− 1.23	0.167	7.35E−13	*RPRD1B*
	chr10:66,637,441	A, G	0.07	− 0.69	0.090	1.56E−13	*CTNNA3*
*m/z* 709.0644, RT 190.3	chr8:47,661,411	A, G	0.07	0.93	0.128	1.80E−12	*SPIDR*
*m/z* 101.5811, RT 100.6	chr10:98,377,943	T, C	0.46	− 0.61	0.065	4.82E−19	PYROXD2**
*m/z* 161.1285, RT 106.1 (N6-Methyl-L-lysine)	chr10:98,377,943	T, C	0.46	− 0.49	0.061	6.98E−15	PYROXD2**
*m/z* 162.1321, RT 106.9 (N6-Methyl-L-lysine)	chr10:98,377,943	T, C	0.46	− 0.54	0.064	3.32E−16	PYROXD2**
*m/z* 175.1442, RT 104.5 (Ne,Ne dimethyllysine)	chr10:98,377,943	T, C	0.46	− 0.55	0.056	6.20E−21	PYROXD2**
*m/z* 188.9574, RT 44.2	chr12:66,599,541	C, T	0.06	− 1.22	0.164	5.69E−13	*GRIP1*
	chr15:88,215,350	A, C	0.15	− 0.84	0.116	2.85E−12	*NTRK3*
	chr21:14,901,801	G, C	0.13	− 0.85	0.117	1.94E−12	*NRIP1*
*m/z* 220.1777, RT 24	chr16:7,273,260	C, T	0.08	− 1.03	0.137	3.45E−13	*RBFOX1*
*m/z* 269.0023, RT 125.5 (fenson)	chr4:40,241,127	G, C	0.06	0.47	0.061	9.20E−14	*RHOH*
*m/z* 283.2741, RT 27.6	chr7:12,354,019	G, A	0.12	0.27	0.037	1.55E−12	*VWDE*
	chr7:72,362,534	A, G	0.07	0.35	0.047	3.02E−13	*CALN1*
*m/z* 300.2167, RT 37.1	chr2:210,210,185	C, T	0.31	0.68	0.077	5.62E−17	ACADL**
*m/z* 781.7483, RT 89.3	chr8:3,674,391	C, T	0.11	0.73	0.093	2.29E−14	*CSMD1*

The features with identified metabolites or high/medium confidence annotations are listed with chemical names. EA, effect allele; OA, other allele; EAF, effect allele frequency; *m/z*, mass-to-charge ratio; and RT, retention time in seconds

*Represented as chromosome:position based on the human reference genome GRCh38

**Previously reported genetically influenced metabotypes in adults

**Fig. 3:**
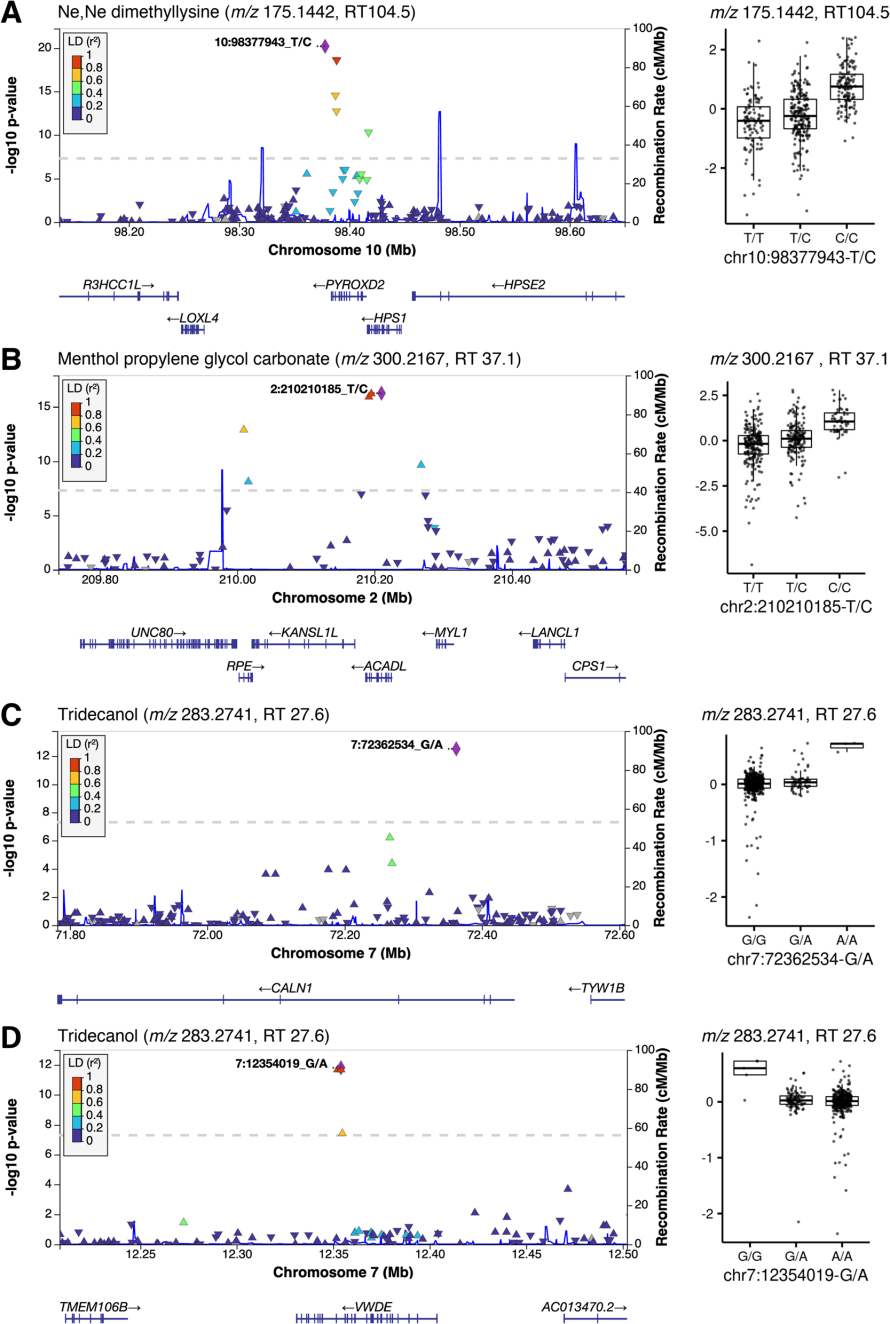
Regional plots for significant genotype–metabotype associations. The genomic coordinates (*x*-axis) are based on GRCh38. The variant with the strongest *p* value is highlighted with its coordinate, reference, and variant alleles. The boxplot (shown next to the regional plot) shows the distribution of normalized feature intensity by genotype for the strongest variant. Feature annotation is from xMSannotator with high and medium confidence or from HMDB with exact mass at 5-ppm tolerance (level 5 annotation according to Schymanski et al.) **A** significant loci for Ne,Ne dimethyllysine (*m/z* 175.1442, RT 104.5) around *PYROXD2* on chromosome 10. **B** loci for Menthol propylene glycol carbonate (*m/z* 300.2167, RT 37.1) on *ACADL* on chromosome 2. **C**, **D** shows the two separate loci on chromosome 7 for Tridecanol (*m/z* 283.2741, RT 27.6), located in two genes *CALN1* and *VWDE*, respectively

A previously reported GIM between *PYROXD2* and Ne,Ne dimethyllysine (*m/z* 175.1442, RT 104.5) was successfully replicated in our pediatric cohort, which was indeed the strongest association (*p* = 6.2 × 10^–21^) in our results (Fig. 3A). This association has been independently replicated by the studies using urine, plasma, and cerebrospinal fluid (CSF) samples [4, 23–25]. The *ACADL* gene encodes acyl-CoA dehydrogenase long chain (ACADL) that is a subunit of the four enzymes involved in the initial step of mitochondrial beta-oxidation of straight-chain fatty acid. One missense and two intronic variants were significantly associated with a feature (*m/z* 300.2167, RT 37.1) that was annotated as menthol propylene glycol carbonate (HMDB identifier: HMDB0039785) at 5-ppm tolerance (i.e., level 5 identification by criteria of Schymanski et al*.* [21]) (Fig. 3B). The association between nonanoyl carnitine and *ACADL* (*p* = 2.3 × 10^–9^) did not pass our stringent statistical significance threshold, while this association has been previously reported.

Lipid species had low *h*^2^ overall; however, we found novel GIM candidates for the features that uniquely matched to lipids based on the level 5 identification criteria of Schymanski et al*.* [21]. Among novel GIMs discovered in our cohort, Tridecanol (*m/z* 283.2741, RT 27.6) level was associated with intronic SNVs in *CALN1* and *VWDE* (*p* values 3.02 × 10^–13^ and 1.55 × 10^–12^, respectively) (Fig. 3C, D). A monostearyl alcohol triglyceride (*m/z* 781.7483, RT 89.3) was associated with an intronic SNV (rs2624100) in the *CSMD1* (CUB and Sushi Multiple Domains 1) gene (*p* = 2.29 × 10^–14^).

Some feature levels were associated with multiple genomic loci. For instance, a feature (*m/z* 188.9574, RT 44.2) was associated with variants in three genes—*GRIP1*, *NTRK3*, and *NRIP1*—on chromosomes 12, 15, and 21 (*p* values 5.69 × 10^–13^, 2.85 × 10^–12^ and 1.94 × 10^–12^, respectively). The *GRIP1* (glutamate receptor-interacting protein 1) gene on chromosome 12q14.3 is involved in synapse formation [26]. The *NTRK3* (neurotrophic receptor tyrosine kinase 3) gene on chromosome 15q25.3 encodes a receptor tyrosine kinase that binds to its ligand neurotrophin-3 and plays a role in nervous system development. AF127577.4 is a long non-coding RNA on chromosome 21q11.2 and 5′-end overlaps with the *NRIP1* gene. Nuclear receptor-interacting protein 1 (NRIP1) is a nuclear protein that interacts with the hormone-dependent nuclear receptors and expressed in neuronal and glial cells [27].

For the identified metabolites, we found several GIM candidates including those previously reported at a less stringent threshold of *p* < 5 × 10^–8^. For amino acids and its derivatives, we found GIM candidates for arginine, glutamate, lysine, and sulfinoalanine. Arginine and lysine levels were associated with multiple genetic loci in different genes. Six loci including intronic variants in the *APBB2* and *CHAF1A* genes were significantly associated with plasma arginine level. Lysine level was associated with four loci including intronic variants in the *CCDC85A, CAMK4, TMEM106B,* and *OSBPL1A* genes. Glutamate is the most abundant excitatory neurotransmitter in CNS, and its plasma level was significantly associated with genetic variants in the *ADAMTS8* and *RHOU* genes. For carbohydrates, glucose level was significant for an intronic variant (rs11954514) in the *HARS1* (Histidyl-tRNA synthetase 1) gene that is a disease-causing gene for Usher syndrome type 3b (MIM ID: 614504) [28]. An intronic variant in the *SLC7A2* gene was associated with plasma glycogen level. The *SLC7A2* gene encodes a cationic amino acid transporter and is implicated in arginine metabolism. Slc7a2 knockout mice had 20% higher blood glucose compared to wild-type mice [29]. *N*-Acetylneuraminate (Neu5Ac) is a sialic acid found in cell membrane. In neuronal cells, Neu5Ac residues are found in membrane bound glycoproteins, i.e., gangliosides. Neu5Ac interacts with bacterial and viral pathogens in diverse cell types. Multiple SNVs in the *PALLD* gene were associated with plasma Neu5Ac levels. Palladin, encoded by the *PALLD* gene, is a cytoskeletal protein found in actin filaments. Among lipid species, butyrylcarnitine-*ACADS* association was notable, which has been reported in independent studies. All associations significant at a threshold of *p* < 5 × 10^–8^, along with *m/z,* RT and annotation, are listed in Additional file 6: Table S5. The full list of annotations with high or medium confidence by xMSannotator is shown in Additional file 7: Table S6.

### Gene-level enrichment with the variants associated with features

Next, we performed a gene-level enrichment analysis for each feature with its GWAS summary statistics using Multi-marker Analysis of GenoMic Annotation (MAGMA) [30]. A total of 572 genes were enriched with variants associated with 217 features at FDR < 0.01 (Additional file 8: Table S7). The most significant association between gene and feature was found for the *SKIDA1* gene and fenson (*m/z* 269.0023, RT 125.5) (FDR 6.64 × 10^–8^). Among the identified metabolites, the *HLA-C* gene was associated with arginine level (FDR 0.0084). Bilirubin was significantly associated with UDP-glucuronosyltransferase (UDP1A) isoforms, which has been replicated by independent studies using different metabolomics platforms [4, 7, 31, 32]. Of the ten features associated with *UGT1A* isoforms, six were identified and/or annotated as bilirubin, while four features were not. Correlation structure of these features showed that the unmatched features could represent other chemicals than bilirubin (Additional file 1: Fig. S5) as UGT1As are the enzymes of the glucuronidation pathway processing small lipophilic molecules such as steroids, bilirubin, hormones, and drugs into water-soluble and excretable metabolites.

**Fig. 4:**
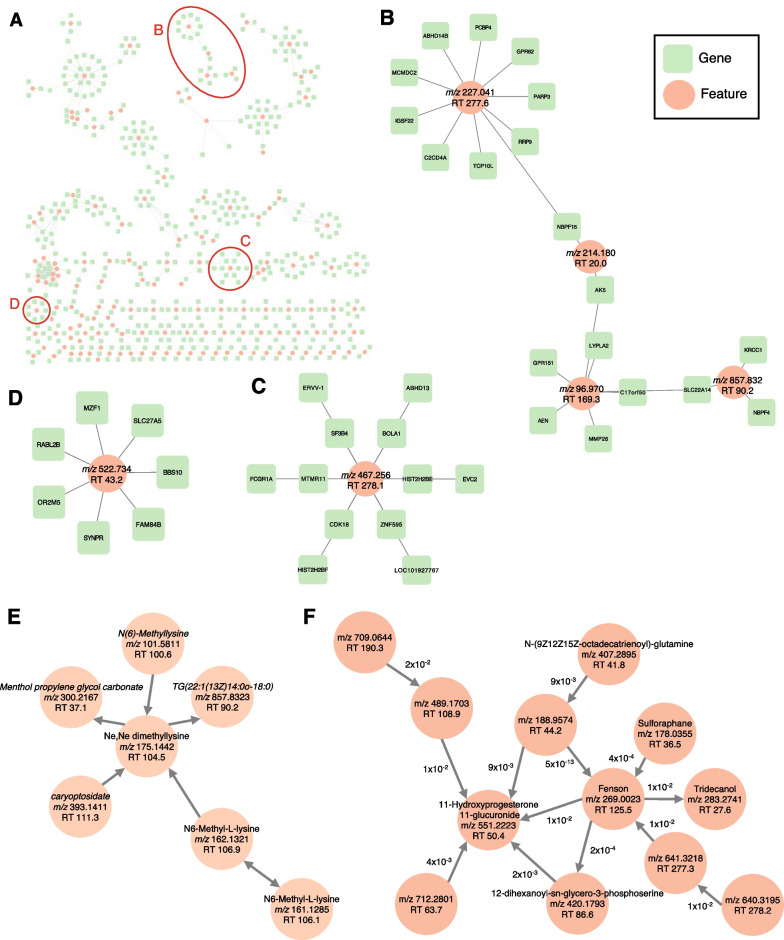
Gene-feature network. The gene-feature network (**A**) with its modules enriched with the Gene Ontology biological pathway terms (**B**–**D**) and causal networks composed of 29 GIMs based on conditional (in)dependency augmented with principles of Mendelian randomization (**E**–**F**). **A** Gene-feature network is constructed using MAGMA results (false discovery rate < 0.01). One-to-one associations are found for 67 gene-feature pairs; however, the other genes and features are interconnected to form modules. **B** A module with four features and the genes enriched with dADP (deoxyadenosine diphosphate) biosynthetic process. **C** Twelve genes associated with a feature (*m/z* 467.256, RT 278.1) are over-represented for nucleosome assembly. **D** Another feature (*m/z* 522.734, RT 43.2) is associated with the seven genes functioning plasma membrane long-chain fatty acid transport. **E**, **F** Two modules identified from the causal network analysis of 29 GIMs. Annotated metabolites in each module provide information about the unannotated metabolites in the same module specially if they are highly connected (e.g., Ne,Ne dimethyllysine (*m/z* 175.144, RT 104.5) in (**E**). In the module in (**F**), a strong dependency (*p* = 5 × 10^–13^) between an unannotated feature (*m/z* 188.9574, RT 44.2) and fenson (*m/z* 269.0023, RT 125.5) may provide annotation information for the feature

We created a network of gene-feature associations with MAGMA results to check the interconnectivity (Fig. 4A and Additional file 1: Fig. S6). The average number of neighbors was 2.0 and a one-to-one association was found for 67 gene-feature pairs. The largest subgraph had 13 features and 72 genes. We checked enriched gene ontology terms for the genes in each subgraph with 6 or more genes. Six subgraphs were enriched with one or more of the Gene Ontology (GO) biological pathway terms (hypergeometric test, FDR < 0.05). A subgraph with 4 features and 19 genes was enriched with the genes involved in purine metabolism such as dADP (deoxyadenosine diphosphate) biosynthetic process (hypergeometric test, FDR 0.018, Fig. 4B). A feature (*m/z* 467.256, RT 278.1) was associated with the variants in 12 genes that were enriched in nucleosome assembly (hypergeometric test, FDR 0.013, Fig. 4C). Seven genes associated with a feature (*m/z* 522.734, RT 43.2) were enriched for plasma membrane long-chain fatty acid transport and ketone body biosynthetic process (hypergeometric test, FDR 0.036 and 0.036, respectively, Fig. 4D).

### Revealing the underlying network modules with genetically influenced metabotypes

Focusing on the 29 GIMs with *p* < 3.5 × 10^–12^, we identified a GIM-causal network at the type I error rate of 5%. Briefly, causal networks are based on conditional (in)dependency established in the principles of Mendelian randomization [33]. We found two disjoint modules of interconnected GIMs. The modules comprised 7 and 12 GIMs with directed connections pointing a prediction target in each module (Fig. 4E, F). A prediction target captures the effect from multiple other GIMs in the module, so its concentration levels can be representative of the module [33]. In the module with seven features (Fig. 4E), menthol propylene glycol carbonate (*m/z* 300.2167, RT 37.1) showed a significant connectivity/dependency (*p* = 2 × 10^–4^) with Ne,Ne dimethyllysine (*m/z* 175.1442, RT 104.5). In the module with 12 features, the connection between an unannotated feature (hilic_1914; *m/z* 188.9574, RT 44.2) and fenson (*m/z* 269.0023, RT 125.5) was one of the most significant connectivities (*p* = 5 × 10^–13^) (Fig. 4F).

## Discussion

In the human body, metabolites have diverse biological functions such as the regulation of epigenome, transcription, translation, protein function, and signal transduction. Further, metabolites are indicators of gene–environment interactions. Previous studies reported genotype–metabotype associations to highlight the genetic contribution to metabolite concentrations; however, the extent of GIMs in the human exposome has not yet been known partly due to the limited coverages of chemical space in previous studies. In the current study, we used an untargeted metabolomics platform that provided a snapshot of thousands of metabolites. Age is one of the key demographic factors contributing the development of exposome. In our previous study, age was correlated with 40.4% of metabolites measured in plasma samples [15]. Interestingly, age-correlated features were enriched for xenobiotics but depleted for nucleic acids and its derivatives. In the current study, we aimed to estimate the contribution of genetic factors to plasma concentrations of diverse chemical species for generally healthy individuals from 5 months to 60 years of age, while most of our study cohort (82.3%) were 20 years old or younger. The genetic contribution to metabolite levels varied across chemical classes. Narrow-sense heritability (*h*^2^) was small (< 0.2) for most features suggesting that environment factors might contribute more substantially to the human exposome than genetic factors, overall. For instance, less than 10% of carbohydrates and lipids—likely linked with diet and lifestyle—had *h*^2^ > 0.6 and none of these species had *h*^2^ > 0.8. In contrast, the genetic influence seemed to be larger for some of amino acids and nucleic acids.

We successfully replicated the previous findings (e.g., *UGT1A1* and bilirubin; *PYROXD2* and methyllysines; and *ACADS* and butyrylcarnitine) that were described in adults. The strongest associations were found between lysine derivatives (i.e., methyllysines) and the genetic variants in *PYROXD2.* This association has been replicated in multiple tissue types. Of note, Panyard and colleagues used 672 CSF samples to perform GxMWAS for 378 metabolites of which the most significant association was found between N6-methyllysine and rs2147896 in *PYROXD2* [4]*.* The same association was also discovered in our analysis of plasma metabolome. Indeed, ten out of top 16 GxMWAS results from the CSF study were also replicated in our study. These results suggest that, for IEMs caused by rare genetic variants with large effect, surrogate tissues such as blood can be used to understand the molecular pathophysiology for which the primary affected tissues are difficult to biopsy.

Environmental contribution to plasma levels of carbohydrates and lipids was larger than genetic factors; however, we found strong GIM candidates for these chemical species as well. Tridecanol (*m/z* 283.2741, RT 27.6) and intronic SNVs in the *CALN1* and *VWDE* genes were significantly associated. The *CALN1* (calneuron 1) is a candidate gene for schizophrenia and intelligence, which was discovered in independent GWASs. The function of *VWDE* (von Willebrand factor D and EGF domains) gene product is not yet known while previous GWASs discovered the risk alleles for frontotemporal dementia, depression, and coronary artery diseases in this gene.

A monostearyl alcohol triglyceride (*m/z* 781.7483, RT 89.3) level was associated with the *CSMD1* gene that encodes a transmembrane protein CSMD1. CSMD1 is an inhibitor of the complement component 3 (C3) convertases that produce C3b and C3a-desArg. Of note, the active C3 fragment, C3a-desArg has insulin-like effects and is involved in triglyceride metabolism [34]. C3 knockout mice demonstrate reduced body weight and fat mass [35]. *CSMD1* is reported as a candidate gene for schizophrenia [36].

For carbohydrates, we found a novel association between plasma glucose level and an intronic variant in the *HARS1* gene encoding HARSL. Loss of function mutations in *HARS1* cause peripheral neuropathies that is likely caused by reduced translation efficiency [37, 38]. In patients with diabetes mellitus, peripheral neuropathy is a common complication observed in 6–51% [39].

Reported GIMs are mostly one-to-one relationships between a metabolite level and a genetic variant (or multiple variants in linkage disequilibrium); however, we found subgraphs with interconnected genes and features in our network analysis. Modularity of gene–metabolite network showed the complexity of genetic contributions to metabolic pathways. Using a causal inferential network analysis method, we extracted direct and indirect contributions of genetic factors to plasma metabolite levels. Of note, a triglyceride and methyl lysine showed a significant connectivity, while the role of PYROXD2 in triglyceride metabolism is not yet known.

Our study had some limitations. Firstly, our sample size (*N* = 441) was not sufficient to replicate some of previous findings at the stringent statistical threshold of *p* < 3.5 × 10^–12^ (= 5 × 10^–8^/14,342 features). For instance, the *ACADS* gene encodes short-chain acyl-CoA dehydrogenase (SCAD) and genetic variants in this gene are associated with mitochondrial fatty acid oxidation function. More than 55 mutations in the *ACADS* gene were reported in patients with SCAD deficiency (SCADD) with increased plasma concentration of butyrylcarnitine [40]. In our analysis, butyrylcarnitine level was associated with eight SNPs in upstream, coding, intronic variants of the *ACADS* gene at *p* < 5 × 10^–8^; however, none of these loci passed the threshold *p* < 3.5 × 10^–12^. Secondly, annotation of features measured by LC-HRMS was not complete. A total of 14,342 features were identified with the combination of unique *m/z*, RT, and peak intensity in our plasma samples. To reduce false annotations, we highlighted significant associations among 891 features with high confidence annotations and 2431 features with medium confidence annotations according to xMSannotator in addition to 166 identified metabolites confirmed with authentic standards. xMSannotator integrates correlation structure of measured features in a dataset with multistage clustering and refinement using diverse chemical annotation databases (e.g., KEGG, HMDB, T3DB, and Lipid Maps) to reduce false annotations. Therefore, some interesting features with significant associations require further annotation and identification of chemical compounds. For instance, a feature (*m/z* 283.2741, RT 27.6) that was associated with the *CALN1* and *VWDE* genes was not annotated by xMSannotator with high or medium confidence nor identified with authentic standard. This feature was uniquely mapped to Tridecanol (HMDB identifier: HMDB0013316) with exact mass (*m/z*) at 1.02 ppm tolerance; however, further investigation is required to confirm structure. Lastly, children enrolled for the current study included patients with rare genetic disorder (e.g., cystic fibrosis) and common diseases (e.g., epilepsy, anemia, and diabetes mellitus). Medication history at the time of blood draw for metabolomics profiling was not used.

## Conclusions

In summary, we performed the most comprehensive analysis to date of the plasma metabolome in a pediatric cohort. Our unbiased profiling methods revealed a wide range of genetic contributions to metabolites for different chemical species as well as complex gene–metabolite associations. The developmental trajectory of a biological system is shaped by gene–environment interactions especially in early life course. Environmental exposures of endogenous and exogenous origins modulate health and aging trajectory of an individual, while genetic factors modify the environmental effect. Continuous efforts on the chemical identification of significant features in HRMS experiments and generating paired genomic and metabolomic data in diverse human tissue types across age groups are required to understand gene–environment interaction toward healthy aging trajectories.

## Methods

### Subjects

Individuals were enrolled in the PrecisionLink Biobank for Health Discovery at Boston Children’s Hospital (BCH) from January 2016 to November 2019 [41]. The participants are enrolled throughout the hospital, across diverse clinical settings. Informed consent is obtained from all participants enrolling in the Biobank and provides permission to: (1) access electronic health record (EHR) data for research; (2) collect and use of residual specimens produced as by-products of routine care; and (3) share de-identified data and specimens outside of the institution. We collected 441 plasma samples from 230 females and 211 males with mean ages 15.7 and 14.3 years old, respectively (ranges from 4.8 months to 60.1 years) (Additional file 3: Table S2). The International Classification of Diseases (versions 9 and 10) and SNOMED CT codes were collected for participants from the BCH Cerner EHR database. To comply with the Health Insurance Portability and Accountability Act rules for protected health information, medical record identifiers and personal information were removed from the EHR extracts and universal unique identifiers (UUIDs) were assigned to everyone. All analyses were performed with UUIDs, age at blood collection, gender information, and sample identifiers for plasma and DNA samples, which were provided by the BCH Biobank. The study was reviewed and approved by the BCH Institutional Review Board.

### Plasma and genomic DNA samples

Genomic DNA (gDNA) and plasma samples were obtained from the PrecisionLink Biobank at Boston Children’s Hospital (BCH). Participants are given the opportunity to also consent to collection of a 4 mL blood sample for research use, from which DNA and plasma aliquots are obtained. In conjunction with other scheduled clinical laboratories, the whole blood is collected from participants in EDTA treated tubes. When received in the Biobank Core Lab, the blood is centrifuged at 2000 × *g* for 10 min at room temperature. Plasma is then aliquoted into 0.5 mL microcentrifuge tubes and stored at − 80 °C in the Biobank Core Lab facility until requested. gDNA is extracted from the whole blood using Gentra Puregene Extraction Kit (Qiagen Sciences Inc, Germantown, MD) or Chemagic B5k Extraction Kits (PerkinElmer, Waltham, MA) resulting in two 0.225 mL aliquots. DNA samples are stored at − 80 °C until requested for research use at which point they undergo normalization and QC. The PrecisionLink Biobank initiative is approved by the BCH Institutional Review Board (protocol number—P00000159).

### High-resolution metabolomics profiling of plasma samples

Plasma samples were thawed and aliquoted at BCH Biobank for shipping to Emory University in dry ice package. We randomized plasma samples to each batch to balance age and sex between batches of HRMS profiling. Plasma samples were extracted by treating 50 μL aliquots with acetonitrile containing 14 stable isotope internal standards with 100 μL to precipitate proteins. Samples were then equilibrated on ice for 30 min and centrifuged for 10 min at 13,400 rpm at 4 °C. The supernatant was transferred to autosampler vials and kept in a refrigerated autosampler until analysis. Each extract was analyzed in triplicate using a dual column chromatography scheme that includes hydrophilic interaction liquid chromatography (HILIC; XBridge BEH Amide XP HILIC column; Waters, Waltham, MA; 50 × 2.1 mm, 2.5 μm) and reversed phase liquid chromatography (RPLC; C18 column; Higgins Analytical, Mountain View, CA; 50 × 2.1 mm, 2.6 μm). The chromatography was coupled with HRMS in positive (HILIC) and negative electrospray ionization (ESI) modes (RPLC) that enabled an increased coverage of the plasma metabolome. Mass spectral data were collected with a 5-min mobile phase gradient on a Thermo Q-Exactive HF high-resolution mass spectrometer (Thermo Fisher, San Diego, CA) set to collect data from *m/z* of 85 to 1275 at a resolution of 120,000 [17]. Raw data were converted to mzXML using ProteoWizard, and data preprocessing, which included peak detection, noise filtering, peak quantification and alignment, averaging signals of triplicates, peak-matching, and batch effect correction, was completed using apLCMS and xMSanalyzer [42, 43]. Raw and processed metabolomic datasets are deposited in the Metabolomics Workbench [44] (Study_ID: ST002331, Project_ID: PR001495, https://doi.org/10.21228/M8GM6Q).

### Metabolite identification and annotation

All features were annotated using xMSannotator [16], which utilizes a multistage clustering algorithm in order to provide confidence scores for annotated metabolites. Subsequent identities of features were compared to our confirmed library of identified metabolites [17] which utilized co-elution relative to authentic standards and ion dissociation mass spectrometry (level 1 identification by criteria of Schymanski et al*.* [21]) with a tolerance of 5 ppm and 30 s. The remaining annotations with high or medium confidence provided by xMSannotator stages 4 and 5 possessed a M − H/M + H adduct, detected in the negative/positive mode, respectively, and were made using the KEGG (Kyoto Encyclopedia of Genes and Genomes) [45]; HMDB (Human Metabolome Database) [46]; T3DB (Toxin and Toxin Target Database) [47]; and Lipid Maps [48] databases at 5 ppm tolerance [17]. For the significant features without high or medium confidence annotations, we added annotations that had exact mass at a 5-ppm tolerance in the HMDB database (level 5 identification by the criteria of Schymanski et al*.* [21]).

### Genome-wide genotyping

Illumina Global Diversity Array (GDA) was used to genotype 1.83 million variants. This platform is used for the All of US program and includes 0.61 million common variants and 0.68 million rare variants covering diverse race and ethnic groups. More importantly, 0.53 million known clinically implicated variants are included such that GDA and can be used for monitoring genetic risks for various common diseases, pharmacogenomics, and frequently mutated genes in rare disorders and cancer. Frozen gDNA samples were thawed for aliquoting and shipping to the Partners Center for Personalized Genomic Medicine (PCPGM) for genotyping using GDAs. All samples were quantitated using picogreen to assess the concentration of double-stranded DNA. QC of the microarrays was carried out by inspecting the Controls Dashboard within GenomeStudio analysis software (Illumina, San Diego, CA). These controls monitor internal spike-in probes at various points of the process and allow the QC of sample-dependent and sample-independent processes. After validating input of 300 ng to each assay, gDNA was amplified using a whole genome amplification process. After fragmentation of the DNA, the sample is hybridized to 50-mer probes attached to the Infinium BeadChip, stopping one base before the interrogated base. Single base extension was then carried out to incorporate a labeled nucleotide. Dual color (Cy3 and Cy5) staining allowed the nucleotide to be detected by the iSCAN reader (Illumina, San Diego, CA) and was converted to genotype during analysis with GenomeStudio.

### Statistical analysis

Features with high coefficient of variation across triplicate measurements and detected in less than 80% of the samples were removed prior to statistical analysis. Batch effects were corrected using ComBat [49]. The peak intensity values were log-2 transformed and adjusted for age, sex, and batches of LC-HRMS profiling by taking the residual values from generalized linear regression with the covariates. For each feature, individuals that were outside of three standard deviations from residual mean value were excluded to reduce potential spurious associations with rare variants.

Out of genotype data from 453 individuals and 1.82 million variants, we used variants and samples that passed all the following exclusion criteria: (1) variants or samples with missing rate less than 2%, (2) bi-allelic variants in autosome with minor allele frequency of 5% or greater, (3) variants passing Hardy–Weinberg equilibrium test (*p* value threshold of 10^–6^), (4) samples without excess heterozygosity (within 3 standard deviations from average across samples), (5) unrelated samples with pairwise King-robust estimator less than 0.177. The final genotype data used in GxMWAS consisted of 441 samples and 619,688 variants in autosomes (including 1036 indels and 17 tri-allelic SNPs each split into 2 bi-allelic SNPs).

The narrow-sense heritability (*h*^2^) of each feature level was estimated using genomic related matrix (GRM) restricted maximum likelihood (GREML) implemented in genome-wide complex trait analysis (GCTA) [22]. For each feature, we fitted a generalized linear model (--glm) [50] implemented in PLINK 2.00a2.3LM [51] with the dosages of minor alleles as independent variables (additive genetic model) and top 10 PCs as covariates to account for population structure. The gene-level enrichment analysis was done for each feature with its summary statistics using MAGMA version 1.0 [30]. All subsequent statistical analysis was performed using R statistical language (version 4.1.2; R Foundation for Statistical Computing, Vienna, Austria).

### Causal network analysis of significant GIMs

We systematically integrated the 29 GIMs and their genetic determinants to identify the GIM-causal network at level 0.05 using the G-DAG algorithm [33]. The causal Bayesian network is augmented with principles of Mendelian randomization (MR). The MR approach is an instrumental variable (IV) technique to identify causal relationships. The assumptions are.

1. IVs are associated with GIMs.
2. IVs are exogenous variables, not affected by metabolites.
3. IVs do not have pleiotropic effect.

We satisfied the assumption 1 by using the genetic determinants of GIMs that are strongly associated with GIMs. The assumption 2 is satisfied since the genetic variations affect metabolites unidirectionally. The assumption 3 is assessed using conditional independence test embedded in the G-DAG algorithm [33].

The GIM-causal network identified from this approach is represented as a directed graph, where the direction represents the direction of effect.

## Supplementary Information

**Additional file 1. Fig. S1.** Population stratification with the first two principal components for all subjects in the study. **Fig. S2.** Distribution of m/z and RT for features associated with age. **Fig. S3.** Distribution of m/z and RT for features associated with sex. **Fig. S4.** Distribution of m/z and RT for features associated with PC1. **Fig. S5.** Correlation matrix among ten features associated with *UGT1A* and isoforms. **Fig. S6.** Visualization of gene-feature association identified using MAGMA.

**Additional file 2. Table S1.** List of features experimentally identified with authentic standards.

**Additional file 3. Table S2.** Demographic characteristics of the pediatric cohort in the study.

**Additional file 4. Table S3.** Features associated with demographic features (age, sex, and genetic ancestry). Only experimentally identified metabolites are listed.

**Additional file 5. Table S4.** Pathways enriched among features associated with demographic features (age, sex, and PC1) by Mummichog.

**Additional file 6. Table S5.** List of all genome-wide significant variant-feature associations.

**Additional file 7. Table S6.** Feature annotations by xMSannotator with high or medium confidence and with 5-ppm mass tolerance.

**Additional file 8. Table S7.** List of all significant gene-feature associations by MAGMA.

## Data Availability

The metabolomic dataset is available in the Metabolomics Workbench (Study_ID: ST002331, Project_ID: PR001495, https://doi.org/10.21228/M8GM6Q).
