# Supplementary material for: Comprehensive characterization of putative genetic influences on plasma metabolome in a pediatric cohort
Source: Hum Genomics. 2022 Dec 8;16:67. doi: 10.1186/s40246-022-00440-w (PMC9730628; doi:10.1186/s40246-022-00440-w)
Supplement: Supplementary file 1 — Additional file 1. Fig. S1. Population stratification with the first two principal components for all subjects in the study. Fig. S2. Distribution of m/z and RT for features associated with age. Fig. S3. Distribution of m/z and RT for features associated with sex. Fig. S4. Distribution of m/z and RT for features associated with PC1. Fig. S5. Correlation matrix among ten features associated with UGT1A and isoforms. Fig. S6. Visualization of gene-feature association identified using MAGMA. [file 40246_2022_440_MOESM1_ESM.pdf]

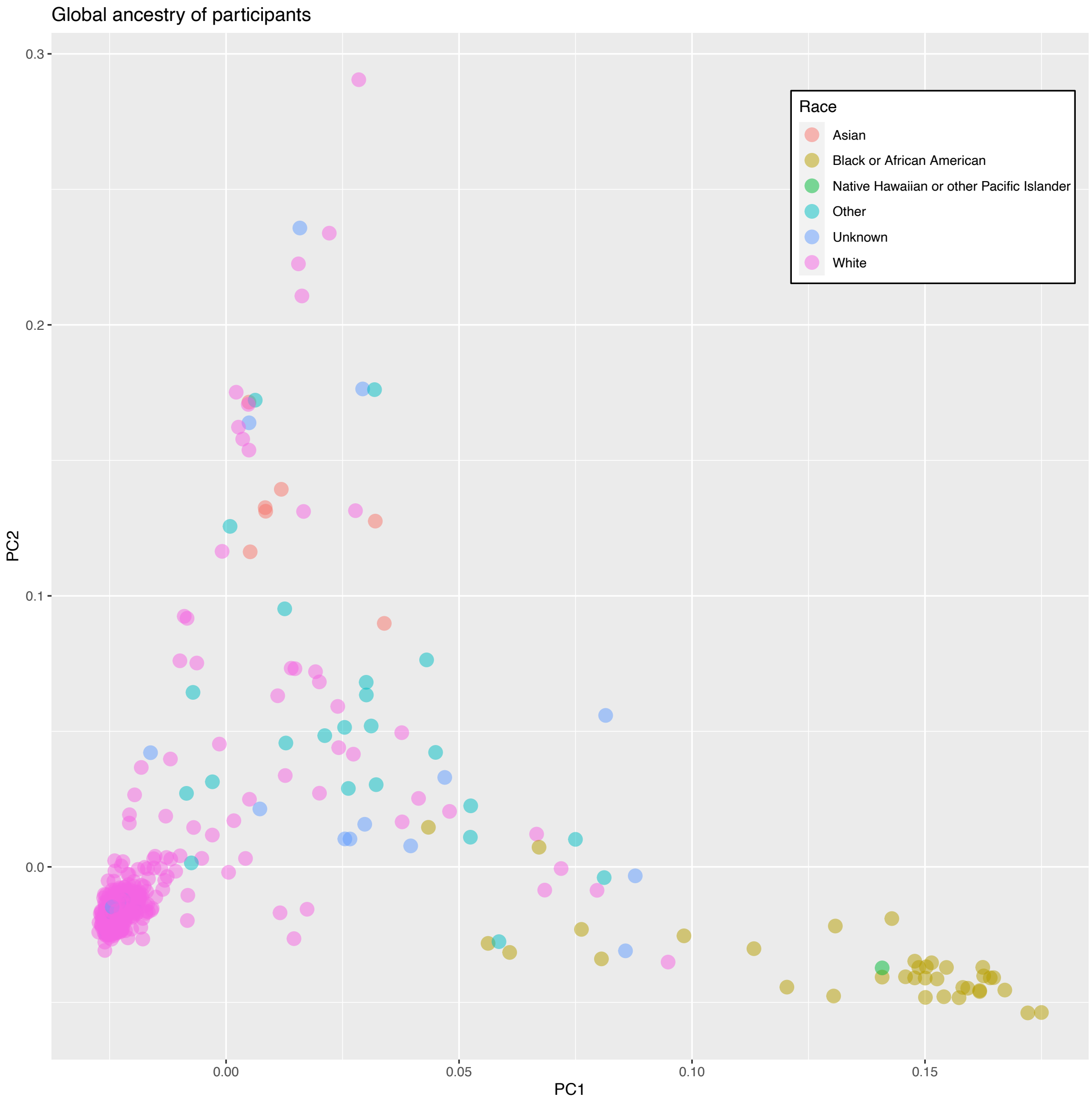

Fig. S1. Population stratification with the first two principal components for all subjects used in the study. Colors represent the self-reported information from participants with terms according to the “Racial and Ethnic Categories and Definitions for NIH Diversity Programs and for Other Reporting Purposes” (https://grants.nih.gov/grants/guide/notice-files/not-od-15-089.html).

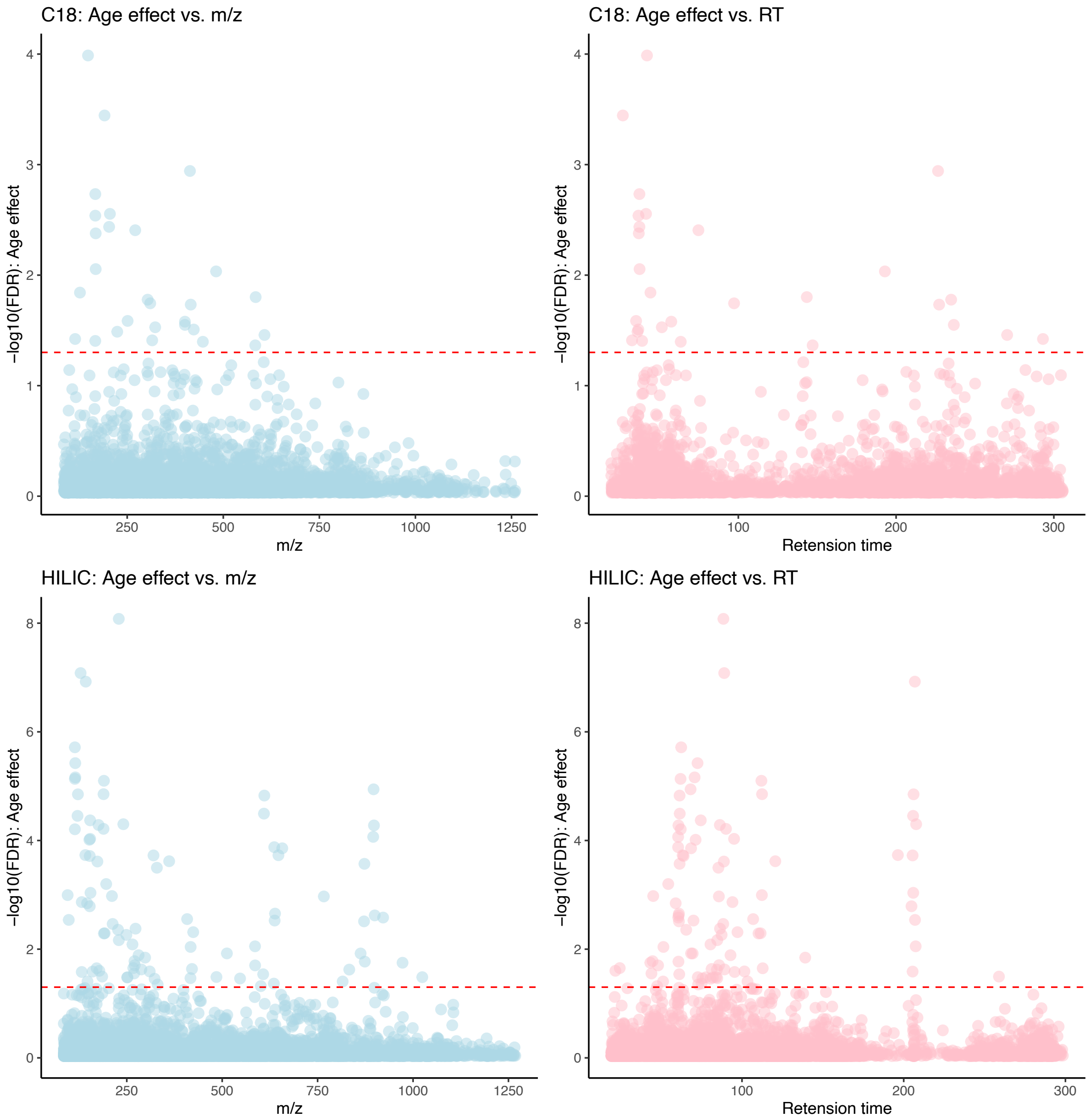

Fig. S2. Distribution of m/z and RT for features associated with age. The dashed horizontal line represent FDR of 5%.

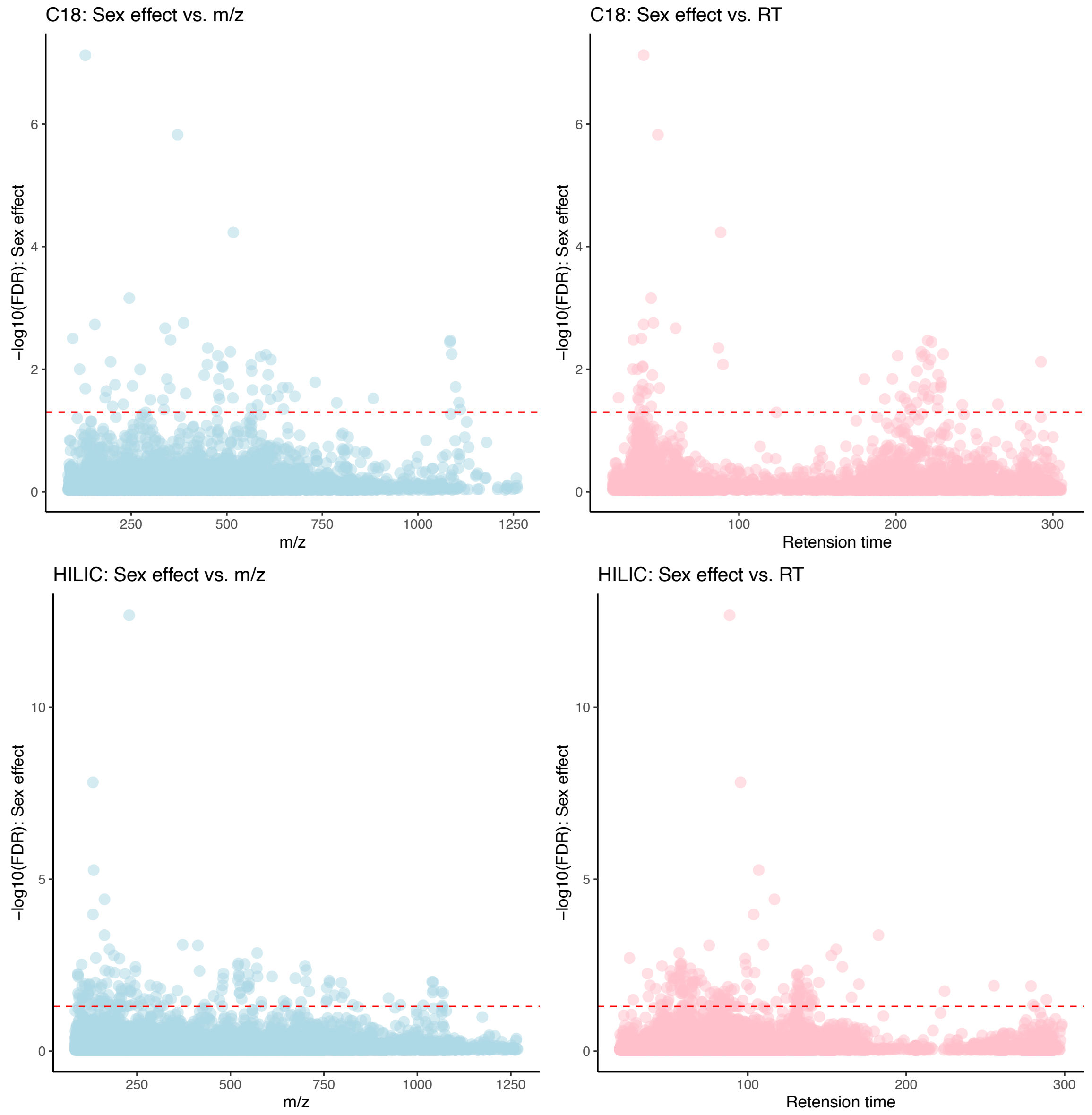

Fig S3. Distribution of m/z and RT for features associated with sex. The dashed horizontal line represent the FDR of 5%.

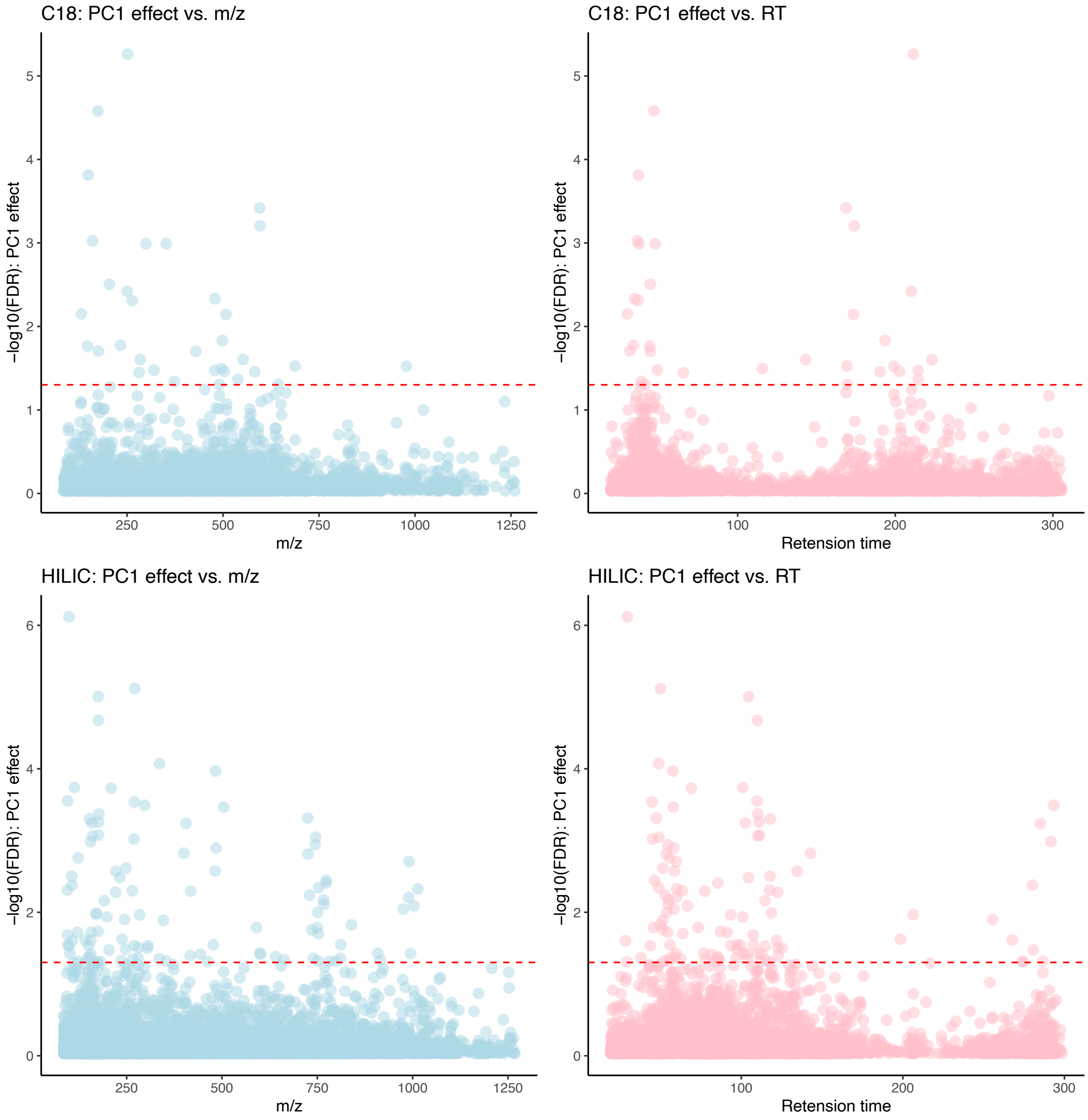

Fig S4. Distribution of m/z and RT for features associated with PC1. The dashed horizontal line represent FDR of 5%.

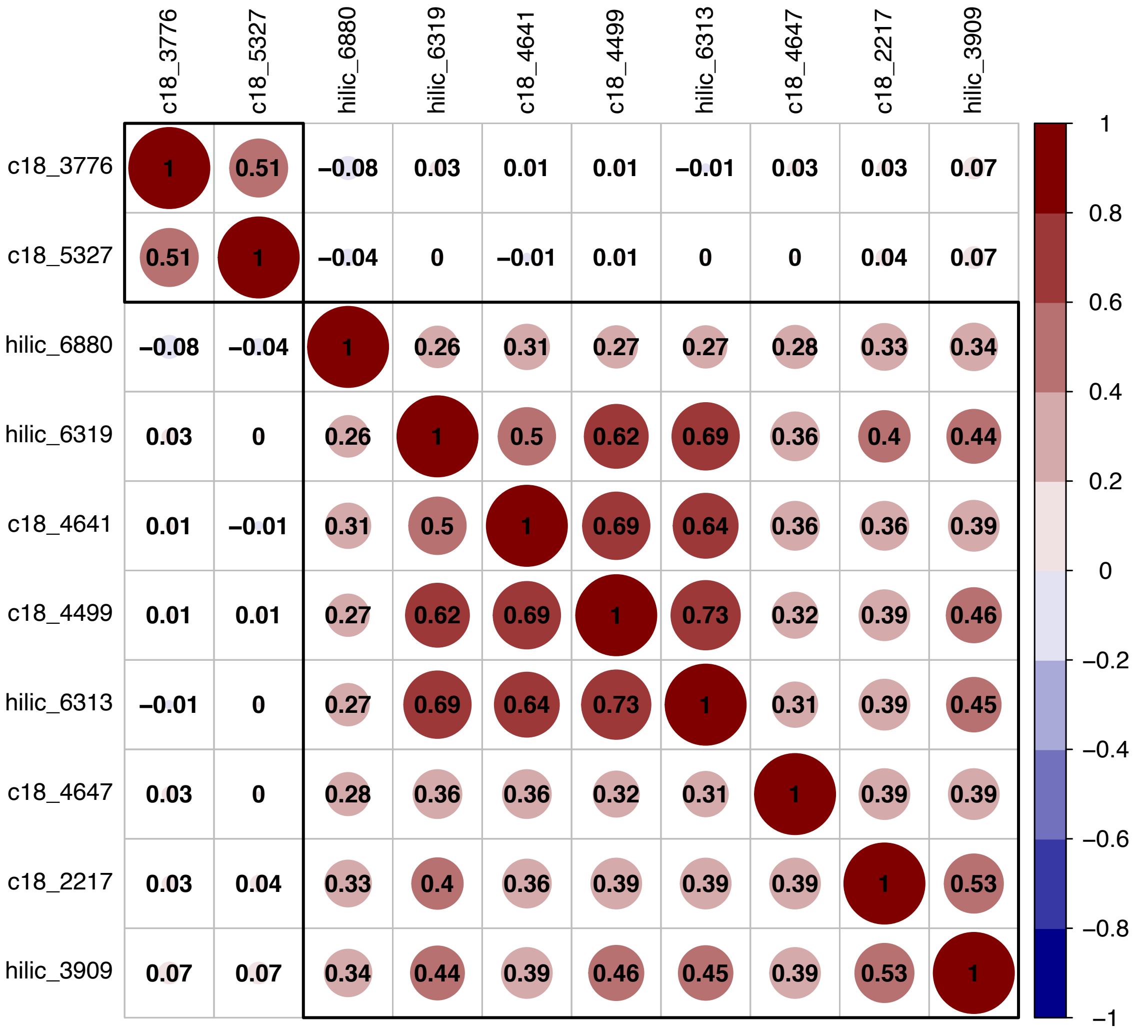

Fig S5. Correlation matrix among the ten features (including features annotated as bilirubin; hilic_6313) associated with *UGT1A* and isoforms. Each cell shows the Pearson correlation coefficient between the corresponding two features, visualized by the size and color of the circle. The clusters among the features are shown as boxes encompassing multiple features.

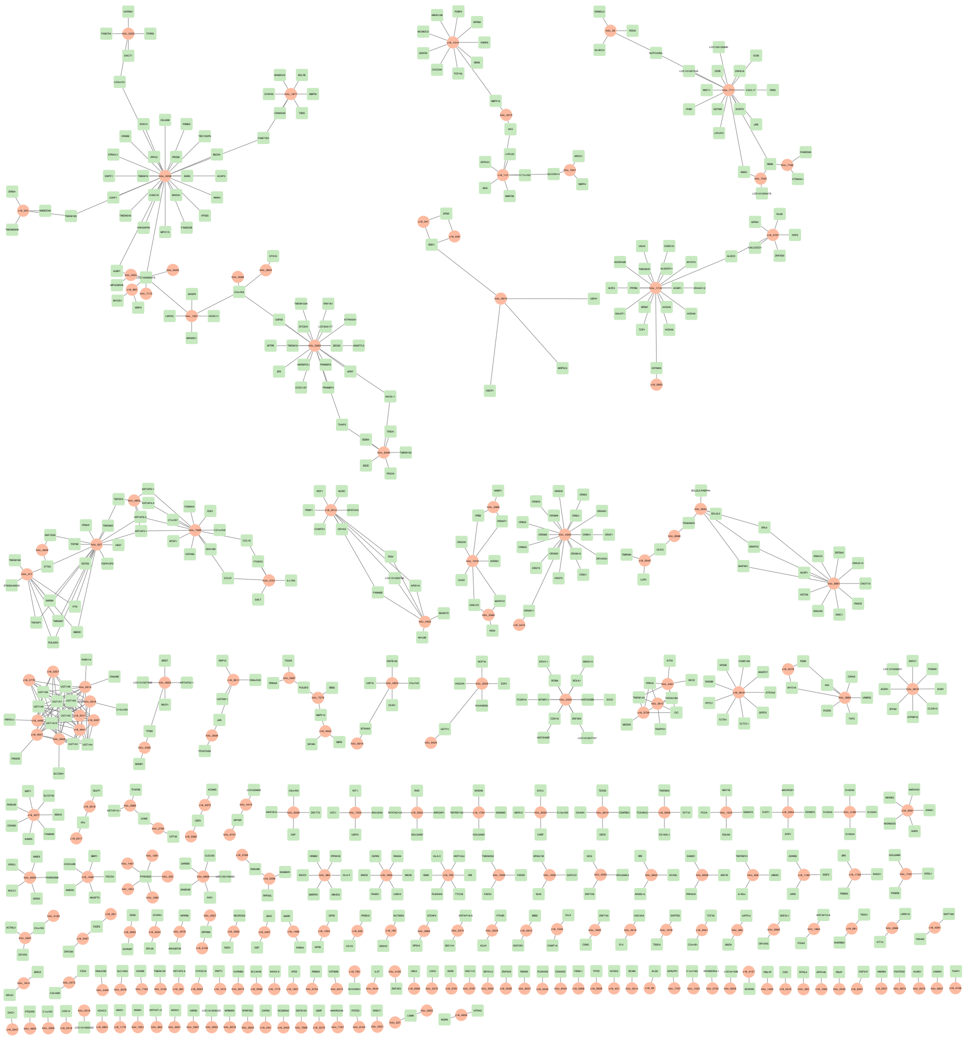

Fig S6. Visualization of gene-feature association identified using MAGMA. The genes and features are represented by circles and squares, respectively. The edges are drawn to connect significant (FDR < 0.01) association between genes and features.
